# Morphine in Strangulated Hernia

**Published:** 1851-05

**Authors:** A. B. Shipman

**Affiliations:** Syracuse, N. Y.


### ARTICLE VI.

Morphine in Strangulated Hernia.—To the Editor of the Boston Medical and Surgical Journal.

Dear Sir,—I have recently met with several cases of strangulated hernia, where reduction was effected by morphine so speedily, with so little pain, and so satisfactorily to the patient, that I am induced to give your readers some brief notes of them from my note book.

In 1841, I published in the New York Medical Gazette several cases of the same nature, and since that time more than three-fourths of the cases of strangulated hernia to which I have been called, were reduced by giving large doses of morphine. An old gentleman, aged 88, in December last was shovelling snow from the side-walk, and a hernia with which he had been affected many years became strangulated. He had been in the habit of returning it without difficulty, but this time he failed, and sent for a physician, who tried all the ordinary means, such as cold applied to the part, nauseating doses of tart. ant., the taxis, &c. &c. Several hours were spent in this manner, but without avail, and I was requested to see him. I found him with a large inguinal hernia, which was tense, tender, and very hard. In addition he had hydrocele of both sides, which were of large size. A large dose of morphine was at once given him, with orders to repeat in an hour if perfect ease was not obtained. Two hours afterwards I saw him. His hips had been elevated according to orders; and I found him in a profound sleep, from which it was difficult to arouse him, yet his hernial tumor had gone back spontaneously, and the use of a truss has since kept it in its proper place.

A young man, 20 years of age, while lifting a heavy weight, felt something give way, and a tumor formed at the external orifice of the abdominal ring. It was very painful, tender and hard. A physician who saw him made use of the taxis, and persevered a long time, but to no purpose. He then gave him a dose of morphine, and advised to send for me. The anodyne which had been given procured some relief, but the taxis was of no avail, the parts being so tender and painful, that it was thought best to desist. A full dose of morphine was now administered, and directions given to elevate his legs and hips. He became easy in an hour and went to sleep. When he awoke, his troubles had ceased; the hernia had spontaneously returned.

Other cases might be recited, which I have seen, or that have occurred in the practice of my medical friends; and so striking has been the benefit of this practice, that, when called early, before inflammation and adhesions of the sac have taken place, I feel an almost certain confidence in its efficacy. The tedious routine of practice recommended by the books, I look upon as worse than useless, in comparison to this. Chloroform is a valuable agent, no doubt; but I have tried it only in two instances, and then with very little hope, as opium had been freely used, and adhesions to the sac were found so firm and extensive, that after the operation of cutting down and separating them, it was found difficult to return the bowel and omentum into the abdomen. I believe that there is scarcely a case of recent strangulation which may not be speedily and easily reduced by a judicious use of large doses of morphine. I have performed but one operation for strangulated hernia, where this practice had been pursued, and failed, except where extensive adhesions existed to account for the failure. That case was one where a large quantity of fæces had been enclosed in the strangulated portion, and also a knot of worms, rendering its return a matter of great difficulty, after I had performed the operation. There is nothing lost if you fail in this course. It gives the patient great ease and comfort, frequently stops the obstinate vomiting, and fits and prepares him for the operation, if that is finally to be performed. The *modus operandi* of the anodyne practice is clearly obvious. When a protrusion of the bowel takes place, pain and irritation at once supervene. Spasm is the next effect, and the opening is now much diminished. Inflammation next follows. Effusion of lymph, and a glueing of the protruded intestine or omentum, or both, to the sac, quickly follows, and prevents its return by taxis or by spontaneous effort. Morphine allays spasm, relaxes the muscles, and allows its return; or it prevents the recurrence of effusion by allaying irritation, and by that means prevents inflammation developing itself, as it would rapidly do were anodynes not exhibited. The practice is not novel, as many writers on surgery recommend opium in the catalogue of remedies for strangulated hernia. But no one, to my knowledge, has made it a prominent agent, but only as one of secondary importance. I view it as more efficient than all other remedies together, and as preferable because of the several indications it fulfils. A. B. SHIPMAN.

*Syracuse, N. Y., March* 22, 1851. —*Ibid.*

